# Clinical translation of pluripotent stem cell-based therapies: successes and challenges

**DOI:** 10.1242/dev.202067

**Published:** 2024-04-02

**Authors:** Josefine Rågård Christiansen, Agnete Kirkeby

**Affiliations:** ^1^ Novo Nordisk Foundation Center for Stem Cell Medicine (reNEW), University of Copenhagen, 2200 Copenhagen N, Denmark; ^2^ Department of Neuroscience, University of Copenhagen, 2200 Copenhagen N, Denmark; ^3^ Wallenberg Center for Molecular Medicine, Department of Experimental Medical Science, Lund University, 221 84 Lund, Sweden

**Keywords:** Human pluripotent stem cells, Cell therapy, Transplantation, Clinical trial, ATMP

## Abstract

The translational stem cell research field has progressed immensely in the past decade. Development and refinement of differentiation protocols now allows the generation of a range of cell types, such as pancreatic β-cells and dopaminergic neurons, from human pluripotent stem cells (hPSCs) in an efficient and good manufacturing practice-compliant fashion. This has led to the initiation of several clinical trials using hPSC-derived cells to replace lost or dysfunctional cells, demonstrating evidence of both safety and efficacy. Here, we highlight successes from some of the hPSC-based trials reporting early signs of efficacy and discuss common challenges in clinical translation of cell therapies.

## Teaser

**Summary:** Great advancements have been made in recent years to bring pluripotent stem cell-based therapies to the clinic. This Spotlight highlights promising clinical results and discusses challenges associated with clinical translation.

## Introduction

The first derivation of human embryonic stem cells (hESCs) in 1998 (Thomson et al., 1998) and later the groundbreaking creation of human induced pluripotent cells (hiPSCs) by reprogramming of somatic cells in 2007 (Takahashi et al., 2007) have incited expectations that a novel class of regenerative therapies based on human pluripotent stem cells (hPSCs) could revolutionize the treatment of chronic diseases. hPSCs are characterized by unlimited self-renewal capacity and differentiation potential, thus representing an ideal source for cell-replacement therapies to treat diseases in which specific cell types are dysfunctional or lost. However, it took around two decades of technology development before the hPSC technology matured to a stage where products started entering clinical trials for several diseases and in larger numbers.

Now, the field has reached a stage where clinical reports of short-term safety as well as early evidence of efficacy are beginning to emerge (Table 1). Prospective confirmation of such results in upcoming phase III studies (see Glossary, Box 1) could indeed be marking the beginning of a new era in regenerative medicine. In this Spotlight, we discuss challenges related to clinical translation of hPSC-based cell therapies and highlight promising advancements in clinical trials.

**Box 1. Glossary**

**Age-related macular degeneration (AMD).** A disease characterized by a loss of central vision. It is caused by a progressive dysfunction and degeneration of cells in the central part of the retina (macula). The dry form is characterized by drusen deposits in the macula, whereas the wet form involves disruption of the RPE layer by penetrating leaky blood vessels from the underlying choroid (Fig. 2) (Bhutto and Lutty, 2012).

**Advanced therapy medicinal product (ATMP).** A medicine that is based on gene, cell or tissue therapy.

**Clinical trial.** Studies intended to evaluate the effects of an investigational medicine in human subjects (Detela and Lodge, 2019). These have three phases: I, II and III. Phase I clinical trials are the first-in-human testing of a medicinal product to evaluate safety. For cell therapies, this is often combined with early evaluation of efficacy and dose-finding in a phase I/IIa trial. Phase II clinical trials evaluate the safety and efficacy endpoints in a larger number of human subjects. Phase III clinical trials confirm clinical efficacy.

**Geographic atrophy.** An advanced form of dry AMD characterized by atrophic lesions in the retina (Bhutto and Lutty, 2012).

**Good laboratory practice (GLP).** A set of rules and criteria concerning the organizational process and the conditions under which non-clinical safety studies are planned, performed, monitored, recorded, reported and archived (European Medicines Agency Good Laboratory Practice Compliance).

**Good manufacturing practice (GMP).** The minimum standard that a medicines manufacturer must meet in their production processes (European Medicines Agency Good Manufacturing Practice).

**Mesial temporal lobe epilepsy (MTLE).** A common form of epilepsy in adults characterized by focal seizures that typically arise in the hippocampus due to imbalanced excitatory and inhibitory neuronal activity (Tatum, 2012).

**Mode of action (MoA).** The process through which a cell therapy product produces its effect.

**Parkinson's disease (PD).** A neurodegenerative disease characterized by a selective loss of dopaminergic neurons in the substantia nigra, resulting in motoric symptoms such as tremor and bradykinesia (Poewe et al., 2017).

**Proof of concept (PoC).** Demonstration that a cell therapy treatment is feasible and efficacious in a certain disease setting. Pre-clinical PoC is typically generated in animal models, while clinical PoC is established in human subjects through phase I and phase II clinical trials.

**Stargardt's macular dystrophy.** An inherited eye disease caused by sequence variants in the *ABCA4* gene. It is the most common pediatric form of macular degeneration (Fujinami et al., 2023).

**Thrombocytopenia.** A condition characterized by an abnormally low number of platelets in the blood, which leads to bleeding complications (Sugimoto et al., 2022b).

**Type 1 diabetes (T1D).** An autoimmune disease in which pancreatic islet β-cells are lost, resulting in insulin deficiency and hyperglycemia (DiMeglio et al., 2018).

**Fig. 1.**
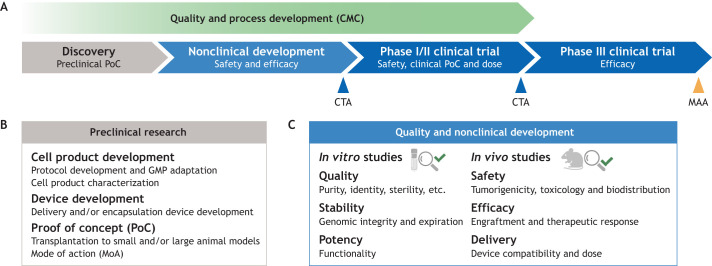
**Steps involved in translating a cell therapy to the clinic.** (A) Schematic representation of the phases from research to market. Following a discovery phase to generate preclinical proof of concept (PoC), the cell product is subjected to nonclinical studies in animal models to demonstrate safety and efficacy, which support the clinical trial authorization (CTA) application. Concurrently, quality and process development, referred to as chemistry, manufacturing and controls (CMC), including raw material qualification, analytical assay development and specification setting, takes place. Upon regulatory approval, the cell product is tested in a small group of patients, typically in a combined phase I/II trial to evaluate safety, dose and provide an early assessment of efficacy (clinical PoC). Clinical efficacy is further evaluated in phase II and phase III trials before applying for market access authorization (MAA) (Detela and Lodge, 2019). (B) The main steps in the preclinical phase. These include development and characterization of the cell product and any necessary devices, as well as transplantation to small (and possibly large) animal models. GMP, good manufacturing practice. (C) The key parameters to be assessed in nonclinical studies. These include the quality, safety and efficacy of the cell product. Created using BioRender.com.

**Fig. 2.**
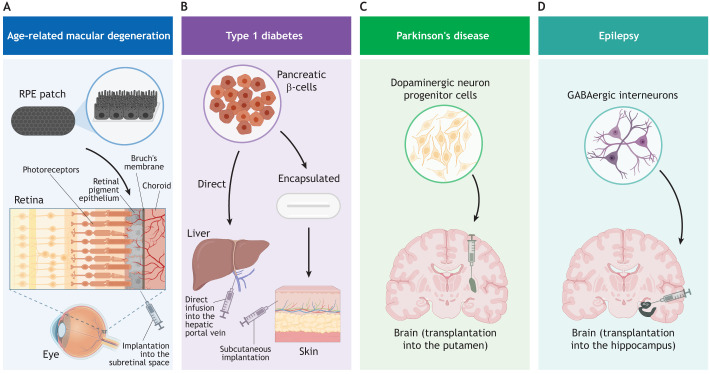
**Illustration of the cell types and injection sites involved in pioneering clinical trials using hPSC-derived cell products.** (A) A patch of retinal pigment epithelium (RPE) on a synthetic membrane is implanted into the subretinal space for treatment of wet age-related macular degeneration (AMD). Inset shows retinal cell layers, including photoreceptors, RPE, Bruch's membrane and choroid. Several clinical trials have been initiated to evaluate cell therapy products for AMD, and not all are covered in this article. Examples of phase I/II clinical trials using the RPE patch approach are NCT01691261 (UK) and NCT02590692 (USA). Other trials involve delivery of RPE-like cells in suspension, e.g. NCT01344993 (USA). (B) Pancreatic β-cells for treatment of type 1 diabetes are administered by direct infusion into the hepatic portal vein or implanted subcutaneously inside an encapsulation device. Phase I/II trials to evaluate these treatment strategies include NCT02239354, NCT04678557, NCT03163511, NCT04786262 and NCT05791201 (USA/Canada). (C) Dopaminergic neuron progenitor cells are injected into the putamen to treat Parkinson's disease. Clinical evaluation is ongoing in phase I/II trials by several teams: UMIN000033564 (Japan), NCT04802733 (USA/Canada), NCT05635409 (Sweden/UK) and NCT05887466 (South Korea). (D) Inhibitory GABAergic interneurons are transplanted into the hippocampal region for treatment of mesial temporal lobe epilepsy. Clinical evaluation is ongoing in phase I/II trials NCT05135091 (USA). Created using BioRender.com.

**Table 1.**
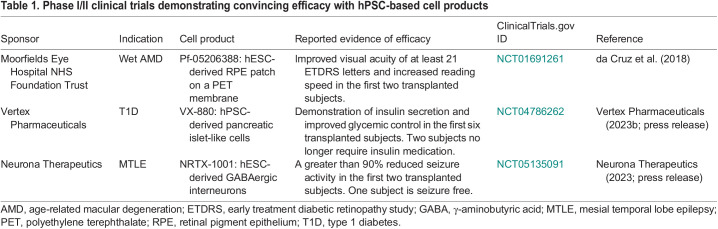
Phase I/II clinical trials demonstrating convincing efficacy with hPSC-based cell products

## Challenges in clinical translation of cell therapies

Progressing hPSC-based cell therapies to clinic has been associated with three main challenges (Armstrong et al., 2020; Barker et al., 2023; Skidmore and Barker, 2023). First, advancing differentiation protocols to a stage where authentic and functional cell types can be produced from hPSCs at high purity without the production of dangerous off-target cells has required years of research in studying and testing factors involved in tissue-specific cell type specification (Karbassi et al., 2020; Kim et al., 2020; Wang et al., 2022). Second, *in vivo* testing of the cells has, in many cases, required the development of novel and challenging animal models combining efficient immunosuppression with a genetic or lesion-induced disease phenotype, and specialized methods for cellular grafting to obtain long-term xenotransplants (Harding et al., 2013), in some cases requiring development of purpose-built devices to deliver the cell product (da Cruz et al., 2018; Goswami et al., 2021). Third, adaptation of all raw materials and manufacturing procedures to good manufacturing practice (GMP; see Glossary, Box 1) and the requirement for long safety studies in animals under good laboratory practice (GLP; see Glossary, Box 1) (Fig. 1) has belated entry into the clinic (Barker et al., 2023). This has been further complicated by an uncertain regulatory pathway for such novel types of therapies. Notably, within the European Union, it has taken manufacturing facilities years to implement the 2007 legislation that increased the regulatory requirements on advanced therapeutic medicinal products (ATMPs; see Glossary, Box 1) (Regulation no. 1394/2007) (Detela and Lodge, 2019; Pizevska et al., 2022). Owing to the complex manufacturing procedures, demanding regulatory approval processes and extraordinary costs of cell therapies, many academic teams have partnered with pharmaceutical or biotechnology companies to pursue clinical trials.

The first approved clinical trial using hPSCs was initiated by Geron Corporation in 2010 (NCT01217008) and involved the transplantation of oligodendrocyte progenitor cells in individuals with spinal cord injury (Manley et al., 2017; Priest et al., 2015). The trial was, however, halted after only 1 year due to financial reasons, and further clinical testing of the product was conducted by Asterias Biotherapeutics, later acquired by Lineage Cell Therapeutics (NCT02302157). Clinical data from these first two trials were only released more than 10 years later and showed no functional improvements beyond the level expected from spontaneous recovery (Fessler et al., 2022; McKenna et al., 2022). The early trial termination and lack of efficacy may suggest that this product was simply not yet ready to go into trial in 2010, and points to the importance of sufficiently characterizing the cell product, optimizing manufacturing and delivery procedures, and establishing robust mode of action (MoA; see Glossary, Box 1), as well as securing adequate funding before initiating a clinical trial. Subsequently, efforts have been made to translate other therapies to the clinic with stronger preclinical data and more solid MoA. In the text that follows, we discuss these efforts and consider their implications for the future of pluripotent stem cell-based therapies.

## Promising advancements in clinical trials

### Clinical trial in individuals with age-related macular degeneration is the first to show clinical efficacy

After the Geron trial, one of the first diseases to be targeted with hPSC-based therapies was age-related macular degeneration (AMD; see Glossary, Box 1) – a leading cause of blindness. AMD is characterized by progressive degeneration of the light-sensitive photoreceptor cells in the retina resulting from damage to the underlying retinal pigment epithelium (RPE) (Fig. 2A). Efforts to transplant autologous RPE patches isolated from the patient's peripheral retina to the degenerating macular region provided proof of concept (PoC; see Glossary, Box 1) of RPE replacement (Binder et al., 2004, 2002). Subsequently, research groups have pursued hPSC-based RPE replacement, leading to several current clinical trials.

Initial safety of hESC-derived RPE transplantation was shown in two parallel clinical trials (NCT01344993 and NCT01345006) conducted by Advanced Cell Technology (later known as the Astellas Institute for Regenerative Medicine). The cell product was generated by spontaneous differentiation in embryoid bodies followed by manual isolation of RPE cells, which were subsequently injected as a cell suspension into the subretinal space (Lu et al., 2009). These relatively simple manufacturing and delivery procedures allowed early progression to the clinic. The clinical studies, initiated in 2011, involved individuals with end-stage dry AMD with geographic atrophy (see Glossary, Box 1) and individuals with Stargardt's macular dystrophy (see Glossary, Box 1), the most common cause of macular degeneration in children. The transplanted cells were well tolerated, with no serious adverse effects related to the grafts, and increased subretinal pigmentation in the treated area, indicative of surviving RPE cells, was observed in most patients. Slight improvements in vision were observed during the first year after transplantation in a small number of treated eyes; however, substantial functional improvements were absent (Schwartz et al., 2012, 2015).

A more advanced hPSC-based RPE cell therapy was brought to clinical testing in 2013 by the London Project to Cure Blindness. The team, led by Pete Coffey, developed a patch consisting of an hESC-derived RPE monolayer on a biocompatible synthetic membrane (Fig. 2A) (da Cruz et al., 2018). Unlike RPE cells in suspension, the patch allowed the delivery of a fully differentiated and polarized epithelial layer, increasing the chances of functional integration into the retina. Furthermore, the team developed a purpose-built surgical device to deliver an intact patch covering almost the entire macula. Upon completion of nonclinical studies in mice and pigs, the product was tested in a cohort of people diagnosed with wet AMD (NCT01691261). Early clinical data from the first two subjects showed the presence of surviving RPE cells across the entire patch at 1 year after the transplantation (da Cruz et al., 2018). Strikingly, both subjects achieved substantially improved visual acuity of 29 and 21 letters on the Early Treatment Diabetic Retinopathy Study (ETDRS) letter chart, and increased reading speed from 1.7 to 82.8 and from 0 to 47.8 words/min, respectively. As such, this was the first trial to show convincing efficacy of a hPSC-based therapy in humans.

A similar patch approach was used in a phase I/II trial (see Glossary, Box 1) conducted by Regenerative Patch Technologies (NCT02590692), involving the transplantation of hESC-derived RPE on a synthetic membrane, this time targeting individuals with advanced dry AMD with geographic atrophy. Early results from the first five subjects showed successful implantation of the patch in four subjects, demonstrating feasibility and short-term safety. Of the treated individuals, one showed an improvement in visual acuity of 17 ETDRS letters, while the remaining subjects showed no substantial improvements (Kashani et al., 2018). Graft survival and safety was confirmed at 2-year follow-up (Kashani et al., 2022).

Together, these studies prove that hPSC-derived RPE cell therapy is indeed feasible and safe. Furthermore, early yet compelling evidence of efficacy from RPE patch transplantation in individuals with wet AMD underlines the unique potential of this treatment to restore vision. The fact that robust improvement in vision was not achieved in individuals with advanced dry AMD is likely associated with the severe degeneration of all retinal layers, including the Bruch's membrane, which underlies and supports the RPE cells (Fig. 2A), at this late stage of disease (Kashani et al., 2022). These pioneering studies therefore illuminate important considerations for future trials regarding the design of the cell product, scaffold and delivery method, as well as the selection of suitable target patient populations.

### Early efficacy reported in individuals with type 1 diabetes receiving β-cell transplants

Another promising area of hPSC-based cell therapy development is the treatment of type 1 diabetes (T1D; see Glossary, Box 1), a chronic autoimmune disease in which pancreatic β-cells that secrete insulin and regulate blood glucose are destroyed (DiMeglio et al., 2018). Transplantation of pancreatic islet tissue from cadaveric donors has been performed for decades, proving that allogeneic islet transplantation is efficacious (Shapiro et al., 2000). Several research teams have worked towards developing protocols to direct the differentiation of pancreatic progenitor cells from hPSCs (D'Amour et al., 2006; Kroon et al., 2008; Rezania et al., 2012). A challenge in the field has been to generate mature and functional β-cells *in vitro*. This was achieved in 2014 by Douglas Melton and colleagues, and verified by additional research groups, who managed to derive glucose-responsive cells capable of secreting insulin and ameliorating hyperglycemia when transplanted into diabetic mice (Pagliuca et al., 2014; Rezania et al., 2014; Russ et al., 2015). Although the ability to generate cells that display key features of bona fide β-cells is a great advancement, the cell products are not exact replicas of human pancreatic islets (Melton, 2021). An evident difference is the cell type composition; current differentiation protocols generate a mixture of pancreatic endocrine and exocrine cells as well as other cell types, including intestinal enterochromaffin-like cells (Veres et al., 2019). Thus, future efforts to improve the cell product will likely focus on adjusting the proportions of endocrine cells (α-, β- and δ-cells) to increase the number of β-cells and remove undesired cell types. Moreover, there are transcriptomic, epigenomic and metabolic differences between hPSC-derived and cadaveric human β-cells *in vitro*, with the former displaying more immature profiles (Hogrebe et al., 2023; Melton, 2021). Further refinements of differentiation protocols to generate more mature and functional β-cells that secrete more insulin per cell, together with improvements in cell survival upon transplantation, would reduce the number of cells needed per patient and thereby also lower the production costs.

Another challenge in clinical translation has been to identify the optimal delivery method to ensure viability and function of the grafted cells, while preventing immune-mediated graft rejection. This has led some companies to develop special encapsulation devices to shield the implanted cells from the immune system (Fig. 2B). In 2014, Viacyte initiated a first-in-human clinical trial (NCT02239354) in which hESC-derived pancreatic progenitor cells were loaded into a cell-impermeable immunoprotective device and delivered subcutaneously to individuals with T1D without immunosuppression. The treatment was reported to be safe and well tolerated, without signs of allo- or autoimmune reaction; however, the trial was suspended due to poor engraftment and cell survival, likely caused by insufficient blood supply (Henry et al., 2018; Viacyte, 2018). A separate trial evaluated transplantation of the cells in a non-immunoprotective encapsulation device, which allowed direct vascularization (NCT03163511). The 1-year follow-up results from this trial confirmed the safety of the treatment, yet engraftment was still relatively poor and no improvements in glycemic control were observed (Ramzy et al., 2021; Shapiro et al., 2021). In a group of subjects who received a higher cell dose, three out of ten subjects showed an increase in plasma C-peptide, indicative of insulin production in the metabolically relevant range (Keymeulen et al., 2023). However, the insulin secretion was insufficient to restore glycemic control, and the β-cell mass found in an explanted device from the individual with the greatest increase in C-peptide amounted to only 3% of all cells in the device, indicating a need for further optimization of the product.

More promising are the reported results from an ongoing trial initiated by Vertex Pharmaceuticals in 2021 with a fully differentiated pancreatic islet cell product (NCT04786262). In this trial, the cells are delivered intrahepatically by direct infusion into the portal vein and thus require chronic administration of immunosuppressants. Positive results from the first six subjects receiving the treatment were reported in a press release; all subjects showed endogenous insulin production with C-peptide levels in the normal range, and the first two subjects have achieved complete independence from insulin medication (Vertex Pharmaceuticals, 2023b). Although the clinical data are yet to be published, the reported efficacy is highly encouraging. Moreover, after Vertex Pharmaceuticals acquired Viacyte in 2022, the company announced the initiation of a clinical trial (NCT05791201) testing the feasibility of delivering fully differentiated islet cells inside a novel immunoprotective encapsulation device (Vertex Pharmaceuticals, 2023a).

### Dopaminergic neuron replacement holds promise for individuals with Parkinson's disease

Parkinson's disease (PD; see Glossary, Box 1) is an ideal target for cell replacement therapy given the selective loss of a single cell type, the A9 dopaminergic neuron, causing impaired motor function (Poewe et al., 2017). Indeed, cell replacement has been attempted for more than three decades using fetal mesencephalic tissue, and these pioneering efforts have provided evidence that dopaminergic neurons can be successfully engrafted and survive long term in the brain, while providing significant symptomatic recovery in some individuals (Barker et al., 2013). However, the heterogenous nature and insufficient availability of fetal tissue limits the clinical relevance of this approach. As a response to this, protocols for directed differentiation of transplantable dopaminergic progenitor cells from hPSCs have been developed in parallel by several groups (Doi et al., 2014; Kirkeby et al., 2012; Kriks et al., 2011). Transplantation studies in rodents have shown that hPSC-derived dopaminergic progenitor cells are functionally equivalent to those obtained from fetal mesencephalic tissue (Grealish et al., 2014), and optogenetic/chemogenetic graft silencing experiments confirmed that behavioral recovery is induced by neuronal activity-dependent dopamine release (Chen et al., 2016; Steinbeck et al., 2015). Over the past decade, these differentiation protocols have been further improved to increase purity and GMP compliance (Kim et al., 2021; Nolbrant et al., 2017), and preclinical studies have confirmed efficacy and graft maturation in rodents, non-human primates and pigs, paving the way for clinical evaluation of these products (Doi et al., 2020; Kikuchi et al., 2017; Kirkeby et al., 2023; Park et al., 2024; Piao et al., 2021). The current protocols generate mesencephalic progenitor cells that, upon transplantation, further differentiate and mature into dopaminergic neurons with A9/A10 marker expression before integrating and innervating the target region of the host brain (i.e. the dorsal striatum) (Kim et al., 2021; Kirkeby et al., 2023). Furthermore, the transplanted cells possess the ability to buffer exogenous dopamine via the dopamine transporter, indicating mature dopamine neuron function (Elabi et al., 2022). Nonetheless, the transplanted progenitor cells also give rise to other neuronal and non-neuronal cell types (Doi et al., 2020; Piao et al., 2021; Tiklová et al., 2020), and efforts to characterize and adjust the proportions of these cells may lead to better products in the future. Likewise, improvements to enhance the survival of the transplanted cells and increase the number of dopaminergic neurons would reduce the required graft volume and perhaps even result in faster functional recovery.

To date, three phase I/II clinical trials have been commenced, all based on intraputamenal transplantation of dopaminergic progenitor cells (Fig. 2C). The first trial, led by Jun Takahashi, was initiated in Japan in 2018 (UMIN000033564) (CiRA, 2018). While the pre-clinical data supporting this trial have been published (Doi et al., 2020), the primary 2-year endpoint of the clinical trial was due by the end of 2023. The second trial, sponsored by BlueRock Therapeutics, evaluates an hESC-derived cell product developed by Lorenz Studer and Viviane Tabar (Piao et al., 2021) in individuals with advanced PD (NCT04802733). The first subject was dosed in 2021, and 1-year safety results from 12 subjects were released at the Movement Disorder Society annual meeting in August 2023, reporting that the therapy was well tolerated and that indications of graft survival were observed by ^18^F-DOPA PET imaging (BlueRock Therapeutics, 2023). The third ongoing trial, STEM-PD (NCT05635409), was initiated by our teams at Lund University and the University of Cambridge, and dosing was commenced at the beginning of 2023 to evaluate dopaminergic progenitor cell transplantation in individuals with moderate PD (Kirkeby et al., 2023). The primary objective of this trial is to demonstrate safety, feasibility and tolerability of the cell product at 12 months, while secondary objectives include assessment of clinical efficacy at 24 and 36 months. In addition to these ongoing trials, a team in South Korea was recently granted approval to initiate clinical testing of a cell therapy in individuals with PD (NCT05887466) for which the pre-clinical data have just been published (Park et al., 2024).

### Interneuron cell therapy alleviates seizures in individuals with epilepsy

Another exciting development in 2023 was the announcement of 1-year clinical results from a novel inhibitory interneuron therapy developed by Neurona Therapeutics for the treatment of mesial temporal lobe epilepsy (MTLE; see Glossary, Box 1). This is a focal epilepsy in which seizures arise from aberrant neuronal hyperactivity in a restricted area of the brain (Tatum, 2012), thereby constituting a potentially suitable target for inhibitory interneuron delivery to dampen seizure activity. Initial proof of reduced seizure activity in experimental models of epilepsy was shown by transplantation of rodent medial ganglionic eminence (MGE) tissue (Baraban et al., 2009; Hunt et al., 2013). Later, differentiation protocols to generate these MGE cells from hPSCs were developed (Maroof et al., 2013; Nicholas et al., 2013), and transplantation studies in rodents established PoC and MoA by demonstrating synaptic integration of grafted interneurons in the host brain and activity-dependent release of the inhibitory neurotransmitter GABA (Cunningham et al., 2014; Upadhya et al., 2019). The team at Neurona Therapeutics has further improved the differentiation procedure, including a sorting step to generate high purity inhibitory pallial interneurons, which are transcriptomically similar to primary human MGE (Bershteyn et al., 2023). Of the two largest subclasses of pallial interneurons, characterized by the expression of somatostatin (SST) and parvalbumin (PV), respectively, the hPSC-derived cells were primarily of SST identity (30-55% of the grafted cells), while PV-expressing cells were rare. The ideal ratio between SST, PV and other subclasses has yet to be elucidated; nonetheless, despite immature electrophysiological and neurochemical properties, the neurons were sufficiently functional to significantly suppress seizures in a dose-dependent manner when transplanted to a mouse model of MTLE (Bershteyn et al., 2023). Preclinical PoC supported a first-in-human clinical trial (NCT05135091) that involves a single injection of hESC-derived inhibitory interneurons into the hippocampal region of individuals with drug-resistant MTLE (Fig. 2D). In a press release, the company announced remarkable results from the first cohort of subjects (Neurona Therapeutics, 2023). The treatment was reported well-tolerated in all five subjects and, furthermore, >95% reduction in seizure activity was achieved in the first two subjects receiving the treatment. Notably, one subject who experienced more than 30 seizures per month pre-transplantation was reported seizure-free from 7 months after the treatment.

### Other ongoing clinical developments and immune-evasive strategies

Other interesting developments for hPSC-based cell therapies, where clinical results are still pending, include regeneration of injured heart tissue in individuals with chronic heart failure. Research pioneered by Charles Murry and Michael Laflamme has provided PoC for cardiomyocyte transplantation in rodent and non-human primate models of myocardial infarction (Chong et al., 2014; Liu et al., 2018). These studies demonstrate engraftment and electrical coupling of hESC-derived cardiomyocytes transplanted into the infarct region, resulting in improved contractile function. Moreover, recent studies have explored promising strategies to prevent graft-induced arrhythmias, which is one of the major hurdles complicating clinical application (Marchiano et al., 2023).

Additionally, with the advent of methods to differentiate hPSC-derived T and natural killer (NK) cells, off-the-shelf hPSC-based immunotherapies hold potential for the treatment of cancer and autoimmune diseases (Cichocki et al., 2020; Themeli et al., 2013). Fate Therapeutics has played a leading role in this area; recently, the company commenced clinical evaluation of a chimeric antigen receptor (CAR) NK cell product incorporating genetic modifications that enhance cytotoxic function and mitigate the potential for NK cell fratricide (NCT05182073) (Cichocki et al., 2022). Furthermore, promising early results have been reported from their first hiPSC-derived CAR T cell clinical trial (NCT04629729) (Fate Therapeutics, 2022). If proven safe and efficacious long-term, these developments hold great promise for next-generation cell-based cancer immunotherapies and may pave the way for regulatory approval of gene-edited hPSC-based cell products.

A common challenge to all of the above-mentioned trials is the risk of host immune-mediated rejection of the allogeneic cell transplant due to mis-matched human leukocyte antigen (HLA) haplotypes, resulting in the need for immunosuppressive treatment. For therapies targeting the brain or eye, temporary or local immunosuppression may be sufficient due to the relatively immune-privileged nature of these organs. However, life-long immunosuppression, which is associated with serious risks, including infections and cancer (Ruiz and Kirk, 2015), remains an issue for other organs. This has led to the pursuit of autologous cell therapies in which hiPSCs are generated from the patient's own cells. Autologous cell transplants have been applied in single cases of PD (Schweitzer et al., 2020), dry AMD (Mandai et al., 2017) and thrombocytopenia (see Glossary, Box 1) (Sugimoto et al., 2022a), and regulatory permission has recently been granted to conduct clinical trials in AMD and PD with autologous cells (Aspen Neuroscience, 2023; National Institutes of Health, 2022). Although this personalized approach is appealing from an immunological perspective, the associated costs and logistic challenges ultimately pose significant hurdles to widespread clinical use. An alternative is the generation of hiPSC HLA haplobanks allowing HLA matching; this approach can result in successful engraftment in the absence of immunosuppression, as shown by recent studies (Sugita et al., 2020; Yoshida et al., 2023). However, a haplobank would need to contain hundreds of hiPSC lines to cover the world's population with sufficient ethnic representation, and is therefore also very challenging to implement in manufacturing (Gourraud et al., 2012; Taylor et al., 2005). A compelling alternative strategy is the generation of universal hPSC lines genetically engineered to escape recognition by the immune system. One approach used by several groups involves depletion of class I and II HLA expression to avoid adaptive immune cell recognition, combined with HLA engineering or immune checkpoint inhibition to prevent a ‘missing self’ cytotoxic response by innate immune cells (Lanza et al., 2019; Meissner et al., 2022). Gene-edited hypoimmune cells have been shown to survive in healthy immunocompetent mice and non-human primates (Harding et al., 2023; Hu et al., 2023); furthermore, PoC has been confirmed in several relevant disease models, e.g. T1D (Gerace et al., 2023; Hu et al., 2024) and blood cancer (Wang et al., 2021).

Excitingly, CRISPR Therapeutics recently announced the initiation of a phase I trial in partnership with Viacyte (NCT05210530), in which the first subject has already been dosed with an immune-evasive pancreatic cell product (CRISPR Therapeutics, 2022). Potential safety concerns related to the use of immune-evasive cells include undetected virus amplification and malignant transformation (Lanza et al., 2019). These issues are to be addressed and could potentially be alleviated by the introduction of a suicide switch that can be activated to kill the cells should they go awry (Liang et al., 2018; Martin et al., 2020).

## Conclusions and perspectives

Great efforts during the past decade to produce specialized cell types from hPSCs at high purity and establish PoC in relevant animal models have finally led the field to a stage where these cells can be evaluated in human subjects for the treatment of various diseases. Phase I/II clinical trials have demonstrated the safety and feasibility of hPSC-based cell therapies, and encouraging evidence of efficacy has been reported in individuals with AMD, T1D and MTLE. These pioneering studies provide important lessons that will instruct future trials to increase their chances of success. The requirement for long-term immunosuppressive treatment is a common hurdle in clinical translation of allogeneic cell therapies for which genetic engineering of hypoimmune cell lines provides a promising path forward. Additional challenges and strategies for next-generation cell therapies revolve around further refining differentiation protocols to increase maturation and purity, as well as improving graft survival and functional integration; the latter could be achieved through combined cell and gene therapy (Gantner et al., 2020) or co-delivery of multiple cell types to allow reconstruction of complex tissues or enhance vascularization (Sun et al., 2020). The continuous advancements of gene editing technologies are also expected to help drive further development of cell therapy products with enhanced functions. These early successes and ongoing clinical developments provide promise for the future of regenerative medicine and lay the groundwork for more cell therapies to enter the clinic in the coming years.
